# Effect of Dietary Selenium and Vitamin E on Ganders’ Response to Semen Collection and Ejaculate Characteristics

**DOI:** 10.1007/s12011-013-9652-5

**Published:** 2013-04-14

**Authors:** Anna Jerysz, Ewa Lukaszewicz

**Affiliations:** Institute of Animal Breeding, Division of Poultry Breeding, Wroclaw University of Environmental and Life Sciences, Chelmonskiego 38a, 51-630 Wroclaw, Poland

**Keywords:** Organic selenium, Vitamin E, Ganders’ reaction, Semen quality, MDA

## Abstract

Compared to other domestic bird species, geese exhibit the lowest reproductive efficiency (poor semen quality, low egg production, and poor fertility and hatchability rates). From an economic perspective, it is a necessity of improve these reproductive traits. Studies have demonstrated that the essential trace element—selenium—plays key roles in testicular development and the maintenance of spermatogenesis. The aim of the present study was to determine the effect of feed supplementation with organic selenium and vitamin E on ganders’ response to manual semen collection and semen quality. Sixteen 3-year-old White Koluda ganders were randomly divided into two groups. The control group was provided commercial feed while the experimental group was provided with the same commercial feed supplemented with selenium (0.3 mg/kg) and vitamin E (100 mg/kg). The response of individual ganders from both groups to manual semen collection and the quality of the semen collected were evaluated. The supplements increased (*P* ≤ 0.05) the frequency and decreased the time interval of a complete ejaculatory response of the ganders to manual semen collections (82.7 % supplement vs. 73.5 % control). Males from the supplemented group had significantly higher (*P* ≤ 0.01; *P* ≤ 0.05) ejaculate volumes, sperm concentrations, and percentages of viable sperm and lower percentages of immature sperm (spermatids). Lipids peroxidation, expressed in terms of the malondialdehyde concentration, was lower (*P* ≤ 0.01) in semen of the supplemented group (0.172 nmol/50 × 10^6^) as compared to the controls (0.320 nmol/50 × 10^6^). Moreover, the duration of the reproductive period of the ganders in the experimental group was 1 week longer. The results show that supplemental dietary selenium and vitamin E improved both the ganders’ response to manual semen collection and semen quality. We conclude that such feed supplementation could lead to greater economic benefits through increased reproductive efficiency within the goose production industry.

## Introduction

White Koluda goose, derived from the wild Greylag (*Anser anser* L.) goose [1], constitutes more than 90 % of goose population in Poland and other European countries. The breeder flocks are usually kept for four seasons (from February through June, each season). However, while semen quality and fertility rates are acceptable at the onset of the breeding season, both gradually decline over the following months [2–4]. This may be a result of depressed semen quality, fewer natural matings, failure of the gander to respond to manual semen collections, or premature gonadal regression [5–7]. Compared to other poultry species, the reproductive efficiency of the domestic goose is poor due to the following: low male to female ratio (usually no more than three to four females per one male), low semen quality, low egg production, and low fertility and hatchability rates. Natural mating frequency and semen quality differs by gander [8]. During the spring months (March–April), 50 to 70 % of the ganders produce good quality ejaculates. This progressively decreases, and until late in the season, only 17–34 % of ganders produce good quality semen [5, 6, 9, 10]. There are few publications addressing the issue of reproductive efficiency in geese. However, it is apparent that any improvement in the gander’s reproductive performance would have a positive impact on goose production.

Selenium and vitamin E are involved in many biochemical and physiological processes in human and animal organisms, including those related to reproduction [11]. Particularly relevant to semen quality is the antioxidant enzyme glutathione peroxidase (GSH-Px), a selenium-dependent enzyme that serves to protect cellular membranes and organelles from peroxidative damages [12]. Glutathione peroxidase assists in the maintenance of testicular function, spermatogenesis, spermatozoa functions [13], as well as testosterone biosynthesis [14]. Studies in mammals have shown that dietary addition of selenium increased male sexual activity manifested by significantly shortening mating and ejaculation times, as well as increased mating frequency [15]. Experiments with broiler breeders and quails indicated that semen quality, including an increase in the percentages of viable sperm and reductions in the percentages of dead and abnormal sperm, can be achieved by feed supplementation with selenium and vitamin E [16–18].

Lipids associated with the sperm plasma membrane serve as a source of energy and are involved in many biochemical processes [19]. However, due to high concentrations of polyunsaturated fatty acids, docosatetraenoic and arachidonic acids in particular [11], the sperm plasma membrane is vulnerable to lipid peroxidation [20]. This process contributes to the loss of cell membrane fluidity and can be an important indicator of reduced sperm fertilizing capacity [21]. Until recently, it was thought that the lipid peroxidation begins during semen storage in vitro [22, 23]. However, recent studies have reported lipid peroxidation in sperm at the time of ejaculation [19, 24], and in the ductus deferens and testes [25]. Therefore, it would be beneficial for the sperm to be protected by an effective antioxidant defense system. The dietary supplementation of selenium and vitamin E to the rooster diet had significant stimulating effect on GSH-Px activity in seminal plasma, spermatozoa, and testes [20, 26, 27].

The aim of the following study is to determine if supplementation of the gander’s feed with selenium and vitamin E will lead to improved reproductive performance. We compared the males’ responses to manual semen collection (ejaculations), and semen quality between ganders fed with a standard diet and the same diet supplemented with selenium and vitamin E.

## Materials and Methods

### Birds and Management

The experiment was conducted with 16 three-year-old White Koluda ganders randomly divided into two groups (eight individuals each). The control group (CG) was fed with a commercial basic feed (Table 1) and experimental group (EG) was fed with the same commercial basic feed supplemented with 0.3 mg/kg selenium (as 300 mg of selenium yeast—Sel-Plex^TM^, Alltech LTD, USA) and 100 mg/kg vitamin E (200 mg/kg of E-50 Adsorbate—Rolimpex S.A). During the entire reproductive cycle, ganders were kept individually in cages (93 × 70 × 85 cm) on straw-lined floor, in unheated housing, under natural temperature and light conditions. Each gander was provided 350–400 g/day of relevant feed and water ad libitum. Periodically, water basins were provided to the ganders for feather cleaning and other behavioral activities.

**Table 1:** Ingredients and chemical composition of the basic feed used for ganders during the entire reproductive season

Ingredients	Units	Content
Barley ground	%	10.0
Maize ground	%	20.0
Triticale ground	%	15.0
Naked oats ground	%	5.0
Oats ground	%	16.5
Soybean meal (42–46 % CP)	%	5.0
Rapeseed meal solvent 00	%	10.0
Wheat bran <9 % CF	%	10.0
Calcium carbonate	%	6.0
Vitfoss PX 2.5 % GN 1 S	%	2.5
Analyzed chemical composition		
Dry matter	g	883.00
Crude protein	g	146.47
Raw fat	g	29.42
Crude fiber	g	51.09
ME	MJ	10.32

Feed analysis was made in National Feed Laboratory in Lublin, Poland; 1 kg of basic feed contained: in international unit—15,607 vitamin A, 1,500 vitamin D_3_; in milligram—41.39 vitamin E, 1,922.4 choline, 6.58 lysine, 2.86 methionine, 6.03 methionine + cystine, 5.37 threonine, 1.76 tryptophan, 116.21 Mn, 1.0 iodine, 10.09 Cu, 315.33 Fe, 90.02 Zn, 0.15 Se; in gram—27.55 Ca, 7.34 P, 5.34 available phosphorus, 1.40 Na

### Evaluation of Ganders’ Response to the Massage

Manual semen collections were performed according to Chelmonska [9], twice a week (3–4 days intervals), from middle of January till the end of May. The following types of responses to manual semen collection were observed [3]: type 1—best, a spontaneous protruding of the copulatory organ and immediate ejaculation of good quality semen in less than 60 s; type 2—good, response as above but requires more than 60 s; type 3—poor, copulatory organ protruded but no ejaculation; and type 4—no response. Only responses 1 and 2 were recognized as positive and desired. In order to avoid any excessive stress which could affect the ganders’ response, as well as a quantity and quality of semen, the location of the semen collections, time of collection, staff members, and manual semen collection method were identical with each collection.

### Semen Evaluation

Single ejaculates were collected into conical glass tubes and initially evaluated visually (color, viscosity, contamination) during collecting. Within each group, good quality semen was pooled by transferring it with an automatic pipette to one glass tube. Semen collection was completed within 15–20 min.

The following characteristics were assessed in the pooled, freshly collected semen: volume, osmotic pressure (Semi-micro Osmometer, Type ML, Knauer), pH (pH meter Type P731), sperm concentration, spermatozoa morphology, and lipid peroxidation expressed by malondialdehyde (MDA) level. The mean volume of a single ejaculate per male was calculated within each group on the basis of the total volume of pooled ejaculates. Semen quality evaluations were conducted with the pooled semen samples.

Sperm concentration was calculated using a hemocytometer (Thoma-Zeiss chamber). The viability and morphology of the sperm were determined using nigrosin–eosin smears. Three hundred sperm per slide were evaluated at ×1,250 magnification under a light microscope (Nikon Eclipse E 100), with LUCIA General computer program. Sperm were counted as viable when they were pearly white, while sperm stained partially or totally with eosin were consider nonviable. Those sperm considered viable were further categorized into six classes: normal (spindle-shaped head with well-marked acrosome and visible tail), bulb head, spiral head, bent neck, acrosome defect, abnormal midpiece, spermatids (immature cells), and sperm with other deformities (coiled tails, lack of tail, etc.) [8]. The results of the morphological examination were expressed as the percentage of particular forms of the 300 sperm originally evaluated. To measure lipid peroxide formation, MDA concentration was determined according to method modified by Partyka et al. [28]. To enhance detection of lipid peroxidation, two vials containing 50 × 10^9^ sperm (blank trial and treatment sample) were incubated in 1 mL EK diluent [2] with 0.25 mL ferrous sulfate (0.2 mM) and 0.25 mL sodium ascorbate (1 mM), for 1 h at 37 °C. After incubation, 1 mL of 15 % trichloroacetic acid (TCA) and 1 mL of 0.375 % thiobarbituric acid (TBA) were added to the treatment sample and 1 mL of 15 % TCA plus 1 mL of distilled water were added to the control sample. A “zero” sample was made with 1 mL of distilled water, 1 mL of TCA, and 1 mL of TBA. All samples were boiled for 10 min at 100 °C in a water bath (MLL 547 AJL Electronic). The precipitate was removed by centrifugation at 5,000 rpm (centrifuge MPW, 337) for 10 min at 4 °C and the absorbance was determined at 532 nm (Spectrophotometer Carry Conc UV–Vis). Malondialdehyde concentration was defined according to following formula:

$$ \begin{array}{*{20}c} {C={{{{A_{\mathrm{sample}}}\text{-} {A_{\mathrm{control}}}}} \left/ {{\varepsilon \times 1\,\mathrm{cm}}} \right.}} \hfill \\ {\left( {\varepsilon =156\,\mathrm{mmo}{{\mathrm{l}}^{-1 }}\times 1\times \mathrm{c}{{\mathrm{m}}^{-1 }}} \right)} \hfill \\ \end{array} $$

### Statistical Analysis

The data obtained were analyzed with ANOVA and Duncan’s multiple range test (Statistica, version 7.1 StatSoft, Inc. Data Analysis Software System). Mean values, standard deviations, and figures were created using Microsoft Excel.

## Results

### Ganders’ Response to the Massage Procedure

**Fig. 1:**
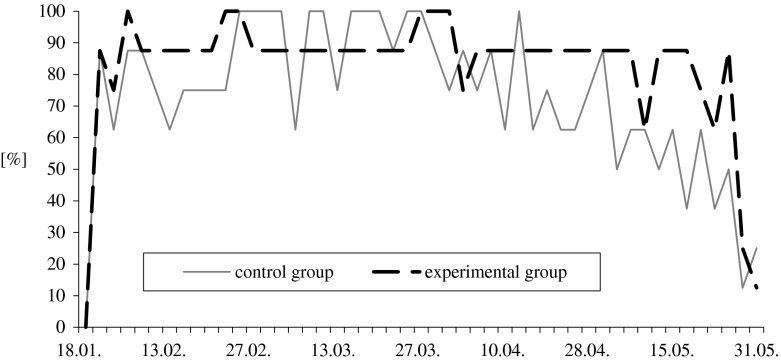
Effect of feed supplementation with organic selenium and vitamin E on percentage of the best responses of White Koluda ganders to semen collection during the entire reproductive season

During the reproductive season (January through May), each gander was subjected to manual semen collection 49 times. Attempts during January were not included in the statistical analysis due to overall poor performance by all ganders. Feed supplementation with organic selenium and vitamin E has a positive effect on the ganders’ responses to manual semen collection and semen quality. Type 1 reactions (erection and ejaculation of good quality semen in less than 60 s) were increased by 12.5 %, while the absence of any response to manual stimulation (type 4) decreased by 60 % when compared to controls (Fig. 1). The most beneficial effect of added antioxidants was observed at the end of the reproductive season when type 1 responses in the experimental group was higher by 42 %, compared to the control group (Table 2).

**Table 2:** Effect of feed supplementation with organic selenium and vitamin E on ganders’ responses (in percent) to semen collection in succeeding months of the reproductive season (means ± SD)

Month of cycle	Best (1)		Good (2)		Poor (3)		No reaction (4)	
	CG	EG	CG	EG	CG	EG	CG	EG
February	79.6a ± 25.2	89.8b ± 7.5	18.8a ± 15.8	6.8b ± 8.6	1.1 ± 4.7	3.4 ± 5.8	1.1 ± 10.7	0.0
March	91.9 ± 12.6	90.2 ± 5.3	8.0 ± 12.6	8.0 ± 6.2	0.0	1.8 ± 4.5	0.0	0.0
April	75.0a ± 13.2	86.3b ± 3.9	21.3A ± 11.9	8.8B ± 6.4	3.8 ± 8.4	3.8 ± 6.0	0.0	1.2 ± 3.9
May	50.0a ± 19.9	70.8b ± 26.3	29.2A ± 16.3	17.7B ± 18.8	7.3 ± 8.4	5.2 ± 6.4	13.5A ± 15.5	6.2B ± 8.4
Entire cycle	73.5a ± 23.7	82.7b ± 19.7	19.1A ± 15.9	9.9B ± 11.7	3.1 ± 6.5	5.6 ± 14.9	4.3 ± 10.7	1.8 ± 5.1

In every group, 392 massages were performed. Mean values in lines within type of reaction with different letters differ significantly: a, b—*P* ≤ 0.05; A, B—*P* ≤ 0.001

*CG* control group, *EG* experimental group

There was a marked division of the gander’s responses to manual collection within each period of the season: responses were higher during February and March and lower during April and May (Table 2). Type 1 and type 2 responses in the control group averaged 83.5 and 13.5 % during February and March and 63.0 and 25.0 % during April and May. In contrast, type 1 and type 2 responses in the experimental group averaged 86.6 and 6.9 % during February and March, and 77.8 and 13.6 % during April and May.

Individuals in both groups of ganders expressed positive responses to manual semen collections, but more constant and intensive susceptibility to this treatment was observed in the experimental group. Seven males (i.e., 87.5 % of the group) in the treatment group elicited type 1 responses in more than 89 % of the semen collection attempts and only two males exhibited type 4 responses. In contrast, only two males in the control group elicited type 1 response in 90 % of the semen collection attempts while three ganders exhibited type 4 responses.

### Semen Quality

**Fig. 2:**
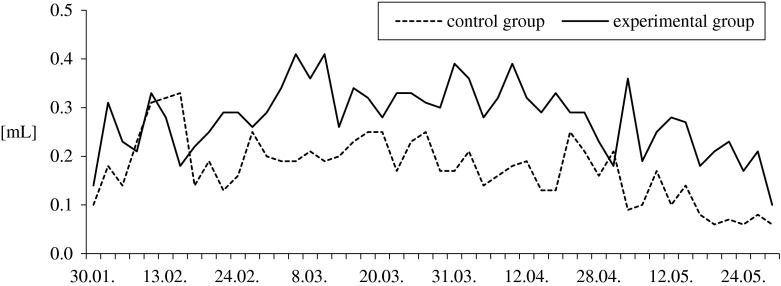
Effect of feed supplementation with organic selenium and vitamin E on the pooled ejaculates volume (in milliliter) of White Koluda ganders during the entire reproductive season

**Fig. 3:**
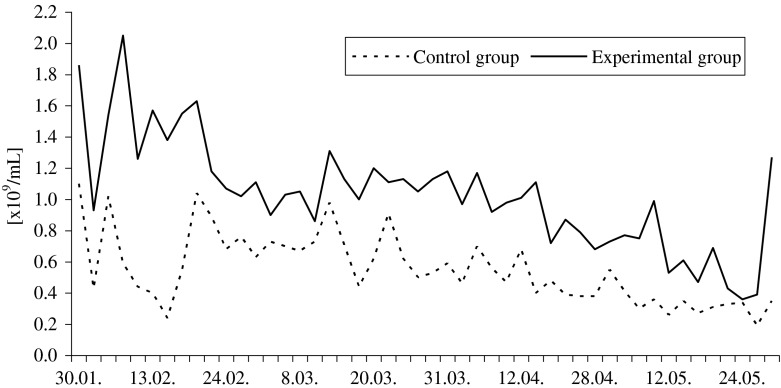
Effect of feed supplementation with organic selenium and vitamin E on the spermatozoa concentration (*n* × 10^9^/mL) in pooled semen of White Koluda ganders during the entire reproductive season

Ganders fed with the supplemented diet had higher ejaculate volumes and sperm concentrations (1.5-fold and 1.7-fold, respectively; *P* ≤ 0.01) throughout the entire reproductive season, compared to the control group ganders. Ejaculate volumes ranged from 0.06 to 0.33 mL in the control group, and from 0.10 to 0.41 mL in the experimental group (Fig. 2). Sperm concentration ranged from 0.19 to 1.10 × 10^9^ mL^−1^ and from 0.36 to 2.05 × 10^9^ mL^−1^, respectively (Fig. 3). Throughout February, March, and April, semen pH in the control group (8.16–8.99) was significantly more alkaline (*P* ≤ 0.05) than in the experimental one (8.11–8.83). Feed supplementation had no effect (*P* > 0.05) on semen osmotic pressure which ranged from 260 to 275 mOsmol/kg in the control and from 260 to 280 mOsmol/kg in the experimental group.

**Fig. 4:**
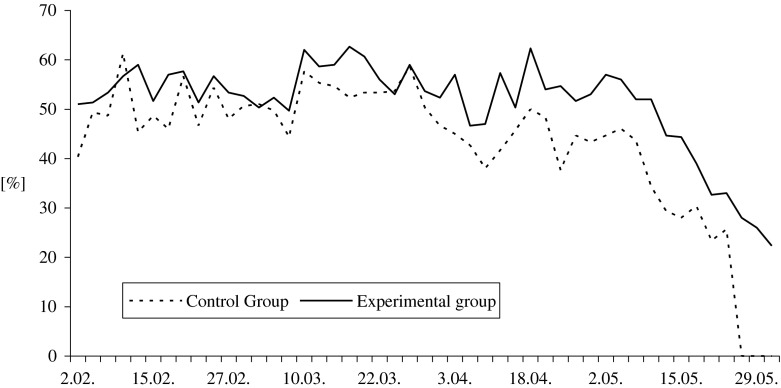
Effect of feed supplementation with organic selenium and vitamin E on the percentage of live normal spermatozoa in White Koluda gander semen during the entire reproductive season

Analysis of sperm morphology showed that the experimental males produced 19 % (*P* ≤ 0.01; *P* ≤ 0.05) more normal sperm (Fig. 4), about 16 % fewer abnormal sperm (*P* ≤ 0.01; *P* ≤ 0.05), and 27 % (*P* ≤ 0.01; *P* ≤ 0.05) fewer spermatids in the semen when compared to the control group (Table 3).

**Table 3:** Effect of feed supplementation with organic selenium and vitamin E on pooled semen characteristics of White Koluda ganders in succeeding months of the reproductive season (means ± SD)

Evaluated traits	Group	Month of the reproductive cycle				Average for season
		February	March	April	May	
No. of collections	*n*	11	14	10	12	
Pooled ejaculates volume [mL]	CG	1.50a ± 0.50	1.46A ± 0.30	1.19A ± 0.21	0.62A ± 0.32	1.2A ± 0.5
	EG	2.07b ± 0.44	2.50B ± 0.37	2.18B ± 0.21	1.40B ± 0.59	2.0B ± 0.6
Ejaculate volume per male [mL]	CG	0.22a ± 0.08	0.21A ± 0.03	0.18A ± 0.04	0.10A ± 0.05	0.2A ± 0.1
	EG	0.26b ± 0.05	0.33B ± 0.05	0.31B ± 0.04	0.22B ± 0.07	0.3B ± 0.1
Sperm concentration [×10^9^ mL^−1^]	CG	0.64A ± 0.26	0.67A ± 0.15	0.49A ± 0.12	0.34A ± 0.09	0.6A ± 0.2
	EG	1.38B ± 0.33	1.09B ± 0.12	0.92B ± 0.16	0.67B ± 0.27	1.0B ± 0.4
Semen pH	CG	8.52a ± 0.16	8.46a ± 0.12	8.53a ± 0.11	8.65 ± 0.17	8.5a ± 0.2
	EG	8.36b ± 0.12	8.32b ± 0.15	8.38b ± 0.12	8.56 ± 0.08	8.4b ± 0.2
Osmotic pressure [mOsmol/kg]	CG	266.7 ± 5.16	269.4 ± 3.20	272.1 ± 3.93	274.0 ± 2.24	270.4 ± 10.1
	EG	271.7 ± 2.58	270.5 ± 3.69	270.0 ± 3.78	275.0 ± 3.16	271.5 ± 3.8
Morphological classes of spermatozoa [%]						
Live normal	CG	49.6a ± 5.8	52.3a ± 3.9	43.7A ± 4.0	25.4A ± 17.0	42.9A ± 14.2
	EG	54.5b ± 2.9	55.9b ± 4.4	53.4B ± 4.8	40.6B ± 12.2	51.1B ± 9.3
Deformed cells in total	CG	42.1a ± 5.5	41.7a ± 3.6	48.9A ± 4.6	65.6A ± 14.3	49.4A ± 12.7
	EG	38.2b ± 2.8	37.7b ± 4.0	39.0B ± 4.1	50.7B ± 10.7	41.4B ± 8.2
Bubble head	CG	13.8 ± 4.1	13.6 ± 2.7	16.3 ± 1.8	40.0A ± 28.7	20.9 ± 18.9
	EG	12.2 ± 2.2	13.8 ± 3.6	15.6 ± 3.8	25.1B ± 9.2	16.7 ± 7.3
Bent neck	CG	6.5 ± 2.1	6.0 ± 1.3	7.1A ± 1.5	4.9A ± 3.3	6.1 ± 2.2
	EG	7.1 ± 2.0	6.5 ± 1.2	5.4B ± 1.0	6.8B ± 1.4	6.5 ± 1.5
Spiral head	CG	1.9 ± 0.7	2.2 ± 0.9	2.0 ± 0.8	1.3a ± 1.16	1.9 ± 0.9
	EG	2.5 ± 1.0	1.7 ± 0.9	1.8 ± 0.6	2.2b ± 0.7	2.0 ± 0.9
Acrosome defects	CG	0.5 ± 0.4	0.8 ± 0.6	0.8 ± 0.5	0.7 ± 0.6	0.7 ± 0.5
	EG	0.5 ± 0.4	0.7 ± 0.3	0.9 ± 0.5	1.1 ± 0.7	0.8 ± 0.5
Changes in midpiece	CG	0.1 ± 0.3	0.2 ± 0.3	0.1a ± 0.2	0.4a ± 0.7	0.2 ± 0.5
	EG	0.1 ± 0.2	0.2 ± 0.4	0.3b ± 0.3	1.2b ± 0.8	0.5 ± 0.7
Other deformities	CG	10.8 ± 2.0	11.7a ± 3.5	12.0A ± 2.0	9.2 ± 6.2	10.9A ± 3.9
	EG	9.4 ± 2.7	8.9b ± 1.7	7.9B ± 1.5	8.0 ± 2.1	8.6B ± 2.1
Spermatids—immature cells	CG	8.6a ± 3.5	7.2a ± 1.7	10.6A ± 1.7	9.0A ± 5.9	8.7A ± 3.7
	EG	6.4b ± 1.6	5.9b ± 1.9	7.1B ± 1.6	6.3B ± 2.2	6.4B ± 1.8
Dead sperm	CG	8.4 ± 4.1	6.0 ± 2.7	7.4 ± 2.4	9.0 ± 3.6	7.6 ± 3.4
	EG	7.5 ± 2.2	6.5 ± 2.7	7.6 ± 4.2	8.7 ± 2.9	7.5 ± 3.0

Mean values in columns, within particular trait, with different letters differ significantly: a, b—*P* ≤ 0.05; A, B—*P* ≤ 0.001

*CG* control group, *EG* experimental group

Among the morphologically abnormal forms of spermatozoa, bulb head was the most frequently observed (Table 3). During the first 3 months of semen production, the frequency of bulb head sperm was similar (*P* > 0.05) for both groups, ranging from 6.0 to 21.0 % in the control and from 6.7 to 23.7 % in the experimental group. However, by May, significant (*P* ≤ 0.01) differences were noted between the groups. In selenium and vitamin E supplemented group, the bulb head sperm ranged from 12.0 to 38.3 % and was approximately 1.6 times smaller than in ganders in the control group (16.3–88.0 %). Also, the total number of abnormal sperm in the experimental group was nearly by 21 % lower (*P* ≤ 0.01; *P* ≤ 0.05) than that observed in the control ganders (Table 3). A similar tendency was noted regarding the presence of spermatids: their percentages varied (*P* ≤ 0.01; *P* ≤ 0.05) from 3.0 to 10.0 and from 3.0 to 16.0 in the experimental and control group, respectively.

**Fig. 5:**
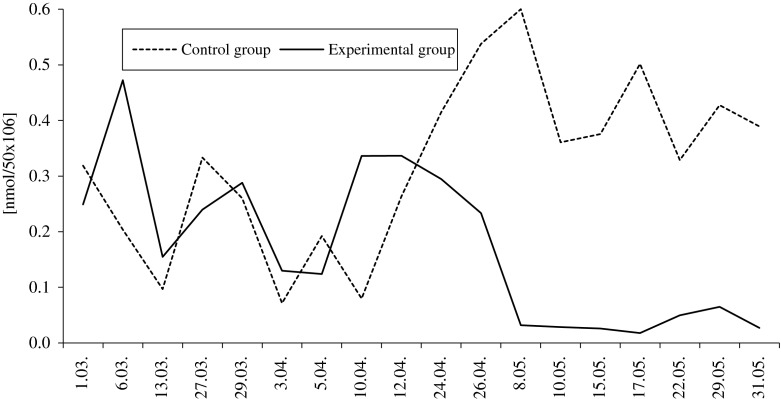
Effect of feed supplementation with organic selenium and vitamin E on the MDA (in nanomoles/50 × 10^6^ cells) concentration in pooled semen of White Koluda ganders during the entire reproductive season

Dietary selenium and vitamin E reduced lipid peroxidation in the collected semen. The average MDA concentration calculated for the entire reproductive season in semen of the experimental group was 50 % less than in the control group. However, throughout the breeding season, the intensity of lipid peroxidation varied between groups, as well as within one group (Fig. 5). The malondialdehyde levels in the semen of the control group ranged from 0.071 to 0.600 nmol/50 × 10^6^ sperm, and in the experimental group from 0.018 to 0.472 nmol/50 × 10^6^. In the first half of the breeding season MDA content was lower (P ≥ 0.05) in the control male semen, but in the second half of the season, significant (*P* ≤ 0.01) differences in MDA levels were observed. In the control group, lipid peroxidation was 12-fold higher than that of the experimental group.

## Discussion

Feeding White Koluda ganders a standard diet supplemented with organic selenium and vitamin E improved their response to manual semen collection and semen quality, and increased their seasonal duration of semen production. The average number of type 1 responses (erection and ejaculation of good quality semen in less than 60 seconds) in the experimental group (83 %) was higher than that observed in 3-year-old ganders (70–75 %) of the same line [3], and 40–50 % observed by Chelmonska [5, 6], Sellier et al. [29], and Varga et al. [10] with 1-year-old ganders of different breeds derived from *A. anser*.

During the first 2 months of the reproductive season, the gander’s frequency of erection and the ejaculation of good quality semen improves and then plateaus at a high level for next 2 months. The gander’s reproductive performance then progressively declines over the last 2 months of its reproductive season [30]. In this study, this pattern was observed only in ganders fed with the basic feed. Ganders fed with the supplemental diet did exhibit a decrease in sexual potency at the end of the breeding season (May–June), while the males of control group were characterized by large fluctuations in the number of type 1 and 2 responses, particularly in second half (April to June) of the reproductive season. Chelmonska [5, 6, 9] observed the best responses of White Italian ganders to manual semen collection in mid-March. There was then a brief decline in mid-April followed by a significant decline in the gander’s response to manual semen collection. In this study, gander’s fed with the supplemented diet maintained type 1 and 2 responses through April, before their responses to manual semen collection began to decline.

Male sexual activity is a function of sex hormones and the proper functional responses of the gonads to the sex hormones [31]. The decline in gander libido is caused by decreases in testicular weight and number of interstitial cells that are responsible for testosterone production [32]. Behne et al. [14] showed that the nuclei of the reproductive cells are able to accumulate selenium which is captured by the interstitial cells and incorporated into testosterone production. The significant and positive effect of antioxidants used in the presented studies on gander susceptibility to sexual stimulation may be associated with testes better utilization of selenium and vitamin E and higher testosterone secretion, which ultimately resulted in extending of the reproductive period by increasing the time gander’s response positively to manual semen collections.

The results of gander semen characteristics also indicate the positive effects of feed supplementation with selenium and vitamin E. In this study, the average ejaculate volumes, sperm concentration, and sperm viability and morphology in ganders fed with antioxidant-enriched feed were higher than ganders fed with the control diet. In addition, the semen characteristics of the ganders fed with the supplemental diet in this study were better than that observed with semen from White Italian [5, 6], White Koluda [33], wild Greylag [34], Cuban [35], and Hungarian geese [10].

In roosters, selenium and vitamin E increased sperm number per ejaculate and improved sperm morphology [16, 17, 36]. Only Gallo et al. [17] observed an increase in the percentage of nonviable sperm. Malaniuk and Lukaszewicz [18] reported a beneficial effect of the two antioxidants on percentage of morphologically normal spermatozoa in Japanese quail semen, but did not observe increased semen volume or sperm concentration. In an experiment performed on goats, Shi et al. [37] reported that selenium increased ejaculate volume, sperm concentration, and sperm motility and reduced, although not significantly, semen pH and the number of morphologically abnormal sperm.

Sperm anomalies observed in the present study were generally similar to those described for other bird species [2, 5, 6, 16, 38, 39]. However, the frequency of occurrence varied. Chelmonska [5, 6], Lukaszewicz [2], and Lukaszewicz et al. [40] reported that head anomalies were the most common. Varga et al. [10] and Alkan et al. [39], in the frizzled Hungarian gander and turkey, respectively, reported that acrosome anomalies were the most frequent sperm anomaly observed. This was not observed with White Koluda gander sperm as acrosomal anomalies were relatively low. Edens and Sefton [16] and Arthur [41] reported that the greatest frequency of sperm anomalies in broiler breeders were concentrated in the midpiece, which is characterized by the array of mitochondria. These authors suggested that when sperm were exposed to a less than optimal environment (i.e., hypotonic medium), the initial damage was at the midpiece. It is speculated that antioxidants may reduce such damage to sperm. Midpiece anomalies in broiler breeder males fed with diets supplemented with selenium went from 36.6 % in the control group to 8.5 % in the group supplemented with sodium selenite, and to 1.0 % when organic selenium was the supplement [16].

In this study, the impact of dietary selenium and vitamin E was observed throughout the reproductive season. Vegi et al. [42] observed a positive effect of selenium and vitamin E on broiler breeder semen quality, including improved sperm motility, sperm concentration, and higher percentages of viable and morphologically normal sperm so in the second half of the breeding season.

A seasonal decline in the gander’s reproductive capabilities is well documented. This may be a result of weakened testes physiological functions. We observed fewer immature testicular sperm (spermatids) in the semen in ganders fed with the supplements when compared to the ganders fed the control diet throughout the production season, thus supporting other observations that show that selenium and vitamin E play an important role in spermatogenesis and sperm maturation [13, 43]. Edens and Sefton [16] indicated that testes weights of 26-week-old roosters of selenium-deficient group were significantly lower than those of selenium-supplemented males. Selenium-enriched roosters had a well-defined hierarchy of spermatogenic cells, and spermatid number in the control group was apparently in greater quantities than in selenium-fed males. This observation has been also confirmed by Marin-Guzman et al. [13] and Behne et al. [14].

Our observations revealed that dietary selenium and vitamin E reduced lipid peroxidation levels in gander semen, particularly in the second half of reproductive season (*P* ≤ 0.01). Other investigations [44, 45] have shown that lipid peroxidation and formation of harmful radicals in poultry spermatozoa increase with the passage of reproductive season. Donoghue and Donoghue [44] reported that at the onset of semen production (30 weeks of age) MDA concentration in tom semen was tenfold lower (0.20 nmol) than after 26 weeks of production (1.37 nmol). Douard et al. [45] stated that in the first half of the reproductive season (between 31 and 41 week of age) there was no change in MDA content in turkey semen, but it increased by 30 % when toms were 47 weeks old and remained constant up to 52 weeks of age. These results are consistent with our control group observations. Malondialdehyde concentrations at the onset of semen production were no different from that observed with the feed supplemented group. However, midway through the reproductive season, MDA concentrations were significantly greater in the control group compared to the feed supplement group. Surai et al. [11, 26] stated that the addition of selenium to the rooster’s diet increased GSH-Px activity in seminal plasma, sperm, and testes, while increased vitamin E concentration in sperm was associated with a reduction in their susceptibility to lipid peroxidation.

The results of discussed study suggest that addition of selenium and vitamin E to the ganders’ diet is beneficial to their reproductive performance. Most likely, the increased levels of these antioxidants contributed to the maintenance of the seminiferous tubules and testosterone biosynthesis that is consistent with observations of Behne et al. [14]. The short goose reproductive season, coupled with their low fecundity, limits the number of goslings obtained and restricts the time of commercial goose production, thus impacting economic profitability [4, 7]. Wang et al. [46] suggested that by increasing the duration the reproductive season of the gander, farm profits would benefit because the price of goslings hatched out of season is several times higher than in-season goslings. The results obtained in this study provide a relatively simple approach to extending the reproductive season of ganders.
